# Contrast Agent Clearance Dynamics and Diffusion in the Mouse Brain After Ventricular Administration

**DOI:** 10.1002/advs.202501502

**Published:** 2025-04-26

**Authors:** Yann Decker, Andreas Müller, Steven T. Proulx

**Affiliations:** ^1^ Department of Neurology Saarland University Medical Center 66421 Homburg Germany; ^2^ Clinic for Diagnostic and Interventional Radiology University of the Saarland 66421 Homburg Germany; ^3^ Theodor Kocher Institute University of Bern Bern 3012 Switzerland

**Keywords:** cerebrospinal fluid, contrast enhanced‐magnetic resonance imaging, diffusion, clearance

## Abstract

Fluid and solute exchange between cerebrospinal fluid (CSF) spaces and the central nervous system (CNS) parenchyma are critical for maintaining neural homeostasis and clearing metabolites. Nevertheless, the pathways and mechanisms underlying fluid and solute exchange between these compartments remain poorly understood. Historically, solute exchange between CSF spaces and the CNS parenchyma has been attributed to diffusion primarily driven by concentration gradients. Recently, the glymphatic hypothesis has challenged this concept by proposing that fluid and solutes move through the brain parenchyma via bulk flow, with influx along arterial perivascular spaces (PVS) and efflux along venous PVS. In this study, we used dynamic contrast‐enhanced MRI to investigate the distribution of two different contrast agents of molecular weights <1 kDa and 17 kDa injected into the lateral ventricle under awake, low‐dose, and high‐dose anesthesia conditions. Our findings revealed an increased CSF bulk flow of both contrast agents from the lateral ventricles to the circle of Willis under awake and low‐dose anesthesia states. In contrast, solute movement into different regions of the brain parenchyma was size‐dependent and affected by the rate of clearance from the ventricles. These observations support the CSF sink hypothesis, emphasizing diffusion‐driven solute exchange as a key mechanism over glymphatic circulation.

## Graphical abstract

Schematic representation of contrast agent clearance by convective bulk flow from the ventricular spaces to the subarachnoid space on the ventral aspect of the brain, or by diffusion into the parenchyma across the pia‐glia or through the semi‐permeable ependyma.

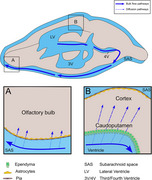

## 1 Introduction

The central nervous system (CNS) requires a tightly regulated fluid environment for the proper functioning of neurons and supporting glial cells and for the clearance of metabolites. The CNS fluids comprise cerebrospinal fluid (CSF) within the ventricles and subarachnoid space (SAS) and interstitial fluid (ISF) in the extracellular spaces (ECS) of the brain and spinal cord parenchyma. However, the mechanisms for fluid and solute exchange between these two fluid compartments are still incompletely understood. Investigators have long considered perivascular spaces (PVS) around the blood vessels to play an important role in this process, with the bulk flow of fluid coupled to solutes proposed to serve as a mechanism for waste removal from the brain.^[^ ^1^, ^2^, ^3^ ^]^

In addition to bulk flow, diffusion through the pial/glia limitans and ependyma has also been repeatedly observed for both small solutes^[^ ^4^, ^5^ ^]^ and larger particles, including serum proteins.^[^ ^6^, ^7^, ^8^ ^]^ Quantification and modeling of tracer spread in the brain parenchyma have demonstrated that diffusion is the dominant mechanism for solute movement within the narrow tortuous spaces of the ECS.^[^ ^9^, ^10^, ^11^ ^]^ Thus, conventional understanding has attributed the exchange of solutes between the CSF spaces and CNS parenchyma to diffusive processes primarily driven by solute concentration gradients. It was proposed that the constant turnover of CSF to the sites of clearance allows this fluid to act as a diffusion “sink” for metabolites in the CNS.^[^ ^12^ ^]^

The glymphatic hypothesis, first proposed in 2012 by Nedergaard and colleagues, has attracted significant attention in the field of neuroscience. Based on two‐photon microscopy observations through a cranial window in mice, the authors proposed a bulk flow of fluid and solutes through the brain parenchyma characterized by influx along arterial PVS and efflux along venous PVS and aided by aquaporin‐4 water channels at astrocytic endfeet.^[^ ^13^ ^]^ Using a 3‐kDa tracer and two‐photon imaging, the Nedergaard group further suggested that glymphatic flow is significantly enhanced during sleep or under anesthesia, facilitating the clearance of neurotoxic waste products, including amyloid beta.^[^ ^14^ ^]^

While the glymphatic hypothesis has gained considerable attention, it has also faced significant objections based on additional experimental evidence or mathematical modeling.^[^ ^9^, ^15^, ^16^, ^17^, ^18^, ^19^ ^]^ Using near‐infrared fluorescence imaging and MRI, we were unable to confirm the penetration of tracers into the brain through perivascular spaces of penetrating arteries while mice were alive.^[^ ^17^ ^]^ Furthermore, the notion that sleep or deep anesthesia drives waste clearance has been further challenged. Using near‐infrared imaging, our findings revealed that following ventricular injection, tracers rapidly exited the subarachnoid space while mice were awake, primarily through lymphatic pathways leading to the systemic circulation. This rapid clearance substantially restricted their distribution to the PVS of the brain.^[^ ^17^ ^]^ Another study employing DCE‐MRI demonstrated that a low molecular weight contrast agent injected into the cisterna magna better penetrates the brain during the awake state.^[^ ^18^ ^]^ More recently, employing fluorescent dye and in vivo photometry techniques, it was shown that brain clearance was significantly reduced during sleep and anesthesia.^[^ ^19^ ^]^

In their seminal work describing the glymphatic hypothesis, Iliff et al. observed perivascular tracer movement following cisternal injections but suggested a lack of significant perivascular and/or interstitial spread when tracers were injected directly into the ventricles.^[^ ^13^ ^]^ This pivotal observation shifted the field toward experimental setups focused almost exclusively on the injection of tracers into the cisterna magna. Furthermore, using DCE‐MRI, Iliff et al.^[^ ^20^ ^]^ visualized significant parenchymal enhancement of a gadolinium contrast agent of <1 kDa but detected only minimal enhancement when using a 200 kDa contrast agent. Since then, the vast majority of investigators using DCE‐MRI to visualize the dynamics of CSF‐infused contrast agents in animal models or humans have employed gadolinium contrast agents smaller than 1 kDa.^[^ ^21^, ^22^, ^23^, ^24^, ^25^ ^]^ Thus, due to the fact that these particles are expected to diffuse readily to the parenchyma and that MRI does not have the spatial resolution to truly render the PVS, it is not possible in these studies to directly attribute contrast enhancement to glymphatic‐mediated mechanisms.

In this study, using DCE‐MRI, we aimed to assess whether the propagation of small‐ and mid‐sized tracers from the CSF spaces to the brain parenchyma aligns with diffusion‐dominated or convection‐mediated mechanisms. Unlike most previous studies, we injected or infused contrast agents directly into the lateral ventricle, close to the source of production of CSF. We used contrast agents of different sizes (<1 and 17 kDa) and investigated their distribution to different regions of the CSF and brain under different physiological conditions (awake, low‐dose isoflurane anesthesia, and high‐dose isoflurane anesthesia). Our data indicates that CSF clearance of contrast agents from the ventricles occurs by a bulk flow process that is enhanced during awake and low‐dose anesthesia conditions, while diffusion of the contrast agent from the ventricles into the parenchyma occurs in a size‐ and concentration‐dependent fashion and is more prevalent during high‐dose anesthesia conditions. Thus, we conclude that solute exchange between the CSF and the parenchyma is consistent with the CSF sink hypothesis rather than glymphatic circulation.

## 2 Results

### 2.1 Clearance of Tracers Injected into the Lateral Ventricle is Increased During Awake and Low‐Dose Isoflurane Conditions

We first aimed to assess the clearance dynamics from the ventricles of two different gadolinium‐based tracers, GadoSpin D (17 kDa) and Dotarem (0.56 kDa), under different vigilance states. Under isoflurane anesthesia, a 33‐gauge stainless steel needle was stereotactically inserted into the right lateral ventricle. Subsequently, 2.5 µL of the chosen contrast agent was injected at a rate of 0.5 µL per min over 5 min. This injection method was selected over low‐rate infusion. A prolonged infusion time would have significantly increased isoflurane exposure for the awake animal group, potentially distorting the physiological state we aimed to replicate.

**Figure 1:**
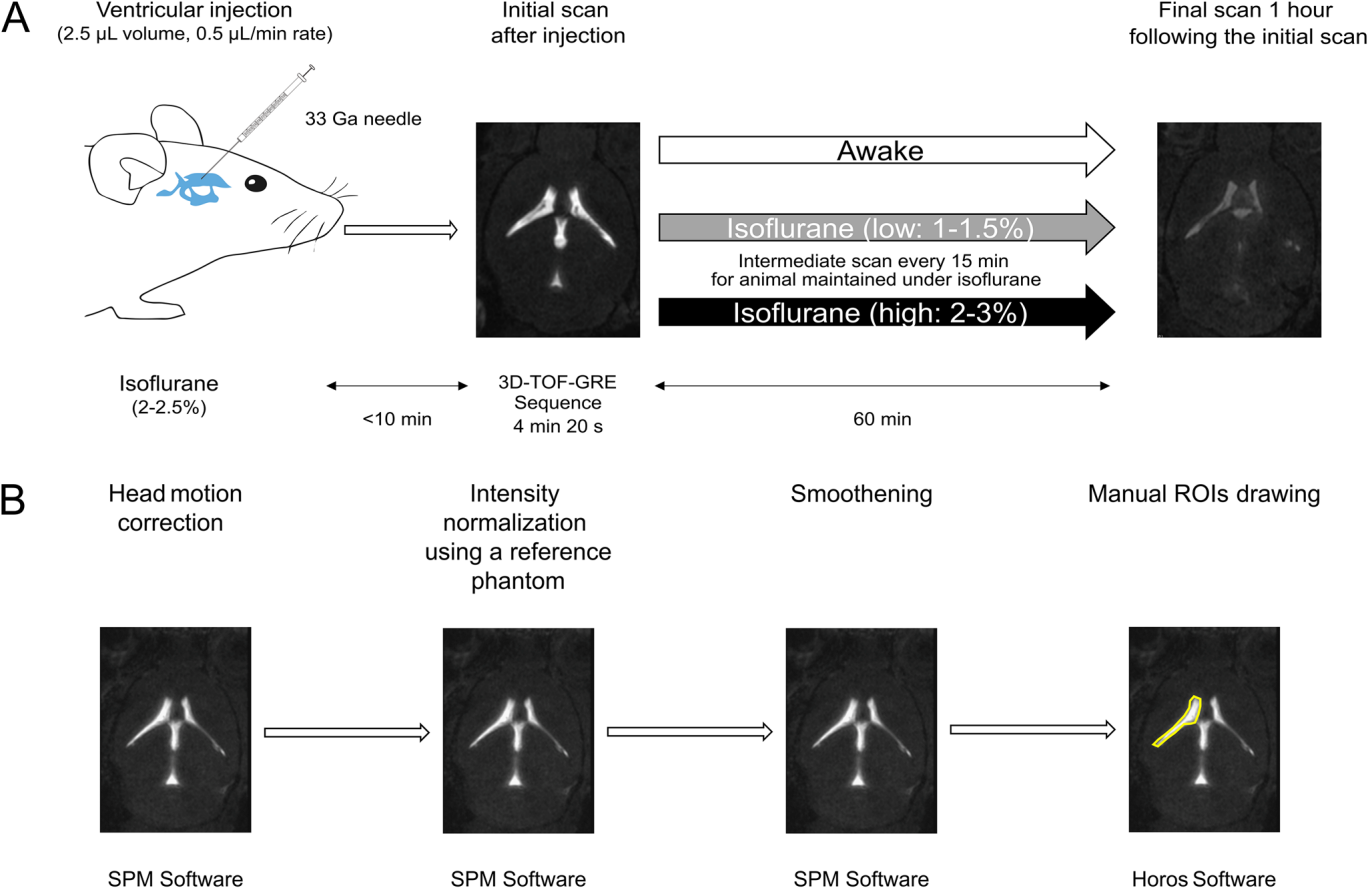
Experimental setup for MRI acquisition and image processing workflow A) Schematic representation of the experimental setup. Mice were injected intraventricularly with either Dotarem (Guerbet, 25 mm Gd) or GadoSpin D (nanoPET Pharma GmbH, 25 mm Gd) at a rate of 0.5 µL per min in a 2.5 µL volume using a 33‐gauge needle. Less than 10 min after injection, an initial T1‐weighted MRI scan was acquired using a 3D time‐of‐flight gradient‐recalled echo (3D‐TOF‐GRE) sequence (scan time: 4 min 20 s). Between the two scans, the experimental conditions were varied, with mice either awake, under low‐dose isoflurane (1–1.5%), or high‐dose isoflurane (2–3%). In the isoflurane low‐ and high‐dose groups, scans were conducted at 15, 30, 45, and 60 min, whereas in the awake group, only a single additional scan was performed at 60 min. Isoflurane anesthesia (2–2.5%) was used before and during the injection of the contrast agent for all mice. B) Image processing workflow. The acquired MRI images were processed using SPM 12 software, which included head motion correction, intensity normalization with a reference phantom, and smoothening. Regions of interest were manually delineated using Horos software (version 4.0.0) to quantify the signal intensity of Dotarem or GadoSpin D in specific brain regions.

After the injection, the mice were immediately positioned prone in an MRI cradle for the 4 min 20 s initial scan. Following the scan, animals either were allowed to recover and move freely in their cages or were maintained on the 37 °C MRI cradle under low‐dose (1–1.5%) or high‐dose (2–3%) isoflurane anesthesia. Animals under isoflurane anesthesia were imaged every 15 min, with the final scan conducted 60 min after the initial scan. In contrast, the awake group was re‐imaged under isoflurane anesthesia only at 60 min after the initial scan (**Figure** **1**A). The acquired MRI images were analyzed using a specific image processing workflow adapted from Iliff et al.^[^ ^20^ ^]^ This included head motion correction, intensity normalization with a reference phantom, and smoothening, all performed using SPM 12 software (https://www.fil.ion.ucl.ac.uk/spm/). Regions of interest (ROIs) were manually delineated in specific brain areas using Horos software to quantify the signal intensity of Dotarem or GadoSpin D (Figure 1B). This experimental setup enabled a precise comparison between the amount of contrast agent initially administered and that present at a given timepoint post‐injection.

**Figure 2:**
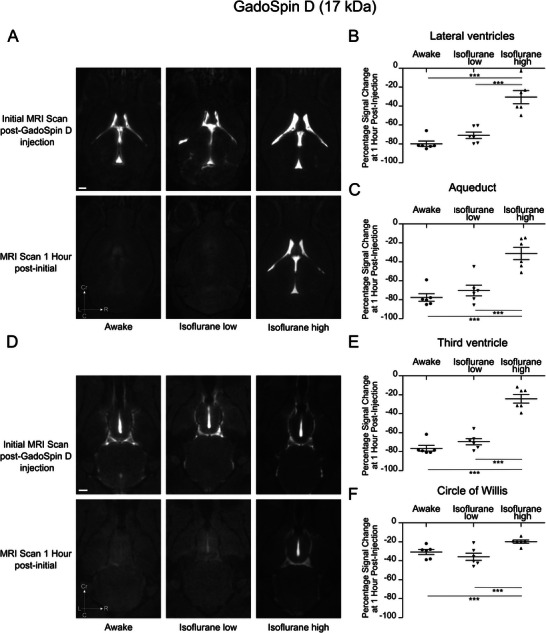
Enhanced clearance of GadoSpin D (17 kDa) from ventricles under awake and low‐dose isoflurane conditions compared to high‐dose isoflurane anesthesia. Representative T1‐weighted MRI axial sections showing tracer distribution following a 2.5 µL intraventricular injection of GadoSpin D (25 mm Gd) at a rate of 0.5 µL per min. A) Scans were acquired using a 3D time‐of‐flight gradient recalled echo sequence with axial sections focusing on the lateral ventricles and cerebral aqueduct. Images were captured <10 min post‐injection and 1 h later following three conditions: awake, low dose (1–1.5%) isoflurane, and high dose (2–3%) isoflurane. Quantification of percentage signal change in the B) lateral ventricles and the C) aqueduct, 1 h after the initial scan across experimental conditions. Additional T1‐weighted axial sections depicting tracer distribution at the level of the third ventricle and the D) circle of Willis, acquired <10 min post‐injection and 1 h later under the same experimental states. Percentage signal change in the E) third ventricle and the F) circle of Willis 1 h after the initial scan across conditions. Data represent *n =* 6 mice per condition. Results are presented as mean ± standard error of the mean. ***p < 0.001 (one‐way ANOVA followed by Tukey's post hoc test). Scale bars: 1 mm.

We first evaluated the distribution of the GadoSpin D tracer from the injection site within the right lateral ventricle to the downstream ventricular system and subarachnoid space. Following injection (**Figure** **2**A), we observed that under both awake and low‐dose isoflurane conditions, the majority of the contrast agent cleared from the lateral ventricle within one hour. Conversely, under high‐dose isoflurane, a substantial portion of the contrast agent remained detectable 1 h post‐injection. Quantitative analysis of the percentage signal change at 1 h (*n =* 6 animals per group) revealed a significant reduction in GadoSpin D signal intensity by ≈70–80% in the lateral ventricles (Figure 2B) and the aqueduct (Figure 2C) for the awake and low‐dose isoflurane groups. In contrast, the high‐dose isoflurane group exhibited only a 30% reduction in signal intensity, indicating significantly greater retention of the contrast agent.

Representative MRI images illustrate that the signal intensity of GadoSpin D in the third ventricle and around the circle of Willis decreased at 1 h post‐injection under awake, low‐dose, and, to a lesser extent, high‐dose isoflurane conditions (Figure 2D). Quantitative analysis of the percentage signal change in the third ventricle 1 h after injection (*n =* 6 animals per group) revealed a significant reduction in GadoSpin D signal intensity of ≈70–76% for the awake and low‐dose isoflurane groups. In contrast, signal intensity in the high‐dose isoflurane group decreased by only 24%, indicating substantially greater tracer retention. A similar trend was observed around the circle of Willis, where signal intensity declined by 30–35% in the awake and low‐dose isoflurane groups. Under high‐dose isoflurane, however, the decrease in signal intensity was markedly smaller, at <20%, further suggesting enhanced retention of GadoSpin D in this region. In summary, we observed that GadoSpin D distribution follows a pathway from the lateral ventricles to the third ventricle and cerebral aqueduct and, ultimately, to the subarachnoid space near the circle of Willis. Clearance occurred at a significantly higher rate across all these regions in mice that were awake and under low‐dose isoflurane compared to those under high‐dose isoflurane alone.

**Figure 3:**
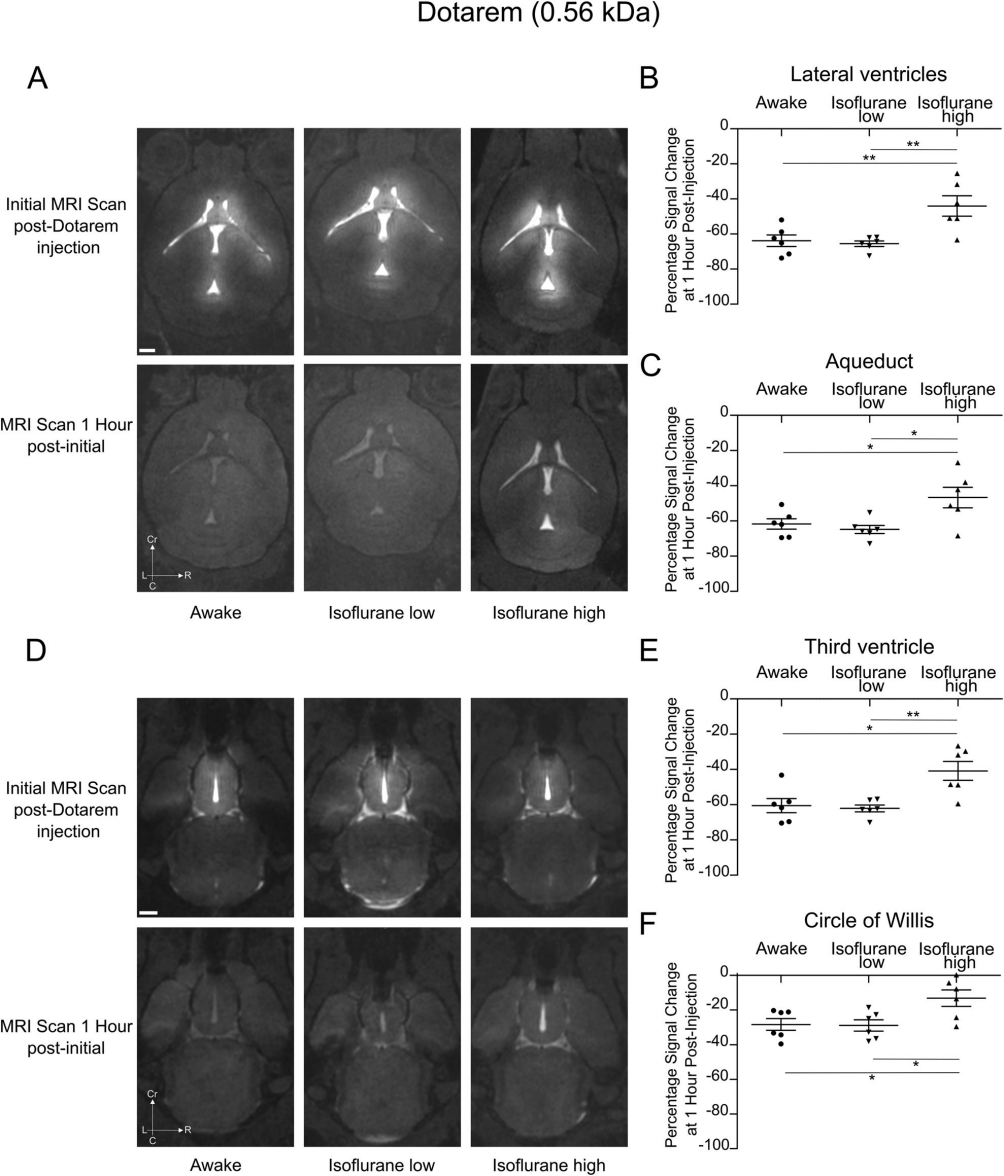
Enhanced Dotarem (0.56 kDa) clearance from the ventricles in awake compared to isoflurane‐anesthetized conditions Representative T1‐weighted MRI axial sections showing tracer distribution following a 2.5 µL intraventricular injection of Dotarem (25 mm Gd) at a rate of 0.5 µL per min. A) Scans were acquired using a 3D time‐of‐flight gradient recalled echo sequence with axial sections focusing on the lateral ventricles and cerebral aqueduct. Images were captured <10 min post‐injection and 1 h later under three conditions: awake, low dose (1–1.5%) isoflurane, and high dose (2–3%) isoflurane. Quantification of percentage signal change in the B) lateral ventricles and the C) aqueduct, 1 h after the initial scan across experimental conditions. Additional T1‐weighted axial sections depicting tracer distribution at the level of the third ventricle and the D) circle of Willis, acquired <10 min post‐injection and 1 h later under the same experimental states. Percentage signal change in the E) third ventricle and the F) circle of Willis 1 h after the initial scan across conditions. Data represent *n =* 6 mice per condition. Results are presented as mean ± standard error of the mean. *p < 0.05; **p < 0.01 (one‐way ANOVA followed by Tukey's post hoc test). Scale bars: 1 mm.

We hypothesized that the transport of the contrast agent from the lateral ventricle to the circle of Willis occurs via a bulk flow, thus molecules smaller than GadoSpin D should exhibit similar clearance patterns from the injection site. To test this, we replaced GadoSpin D with Dotarem, a small molecular weight (0.56 kDa) contrast agent, while maintaining the same experimental conditions. Representative images illustrate the Dotarem clearance from the lateral ventricle and the aqueduct under all physiological conditions examined (**Figure** **3**A). Quantification of the percentage change in signal intensity at these two locations confirmed the general clearance of Dotarem and further revealed a significant clearance in both regions for mice that were awake or under low‐dose isoflurane anesthesia with clearance exceeding 62% (Figure 3B,C). In contrast, mice under high‐dose isoflurane exhibited a lower percentage decrease in signal intensity, of 44–45%. A similar pattern was observed in representative MRI images of the third ventricle and the circle of Willis (Figure 3D). Quantitative analysis further supported these findings, demonstrating significantly greater clearance of Dotarem in both regions for the awake and low‐dose isoflurane groups. In the third ventricle, clearance exceeded 60% for these groups, compared to 39% in the high‐dose isoflurane group (Figure 3E). Similarly, around the circle of Willis, clearance was greater than 27% in awake and low‐dose isoflurane mice, while only ≈14% clearance was observed in the high‐dose isoflurane group (Figure 3F).

Altogether, our observations indicate that two paramagnetic contrast agents, despite differing in molecular weight, are both cleared from the ventricular system to the circle of Willis with consistent clearance rate differences between the physiological conditions. These findings strongly suggest that this process is predominantly driven by CSF bulk flow which is more active during awake or low‐dose anesthetic conditions. However, the % clearance values of the smaller contrast agent during these conditions were consistently lower than the values seen for the larger molecular weight contrast agent. Additionally, we observed that a portion of the smaller contrast agent, beyond being transported to the circle of Willis, was readily apparent in the brain parenchyma, as shown in Figure 3A. This suggests that smaller contrast agents have more access to the brain parenchyma, potentially through passage across the ependymal cells lining the ventricles.

### 2.2 Size‐Dependent Spread of Contrast Agents Through the Parenchyma Supports Diffusion Over Convection

To investigate the diffusion of contrast agents through the brain parenchyma, we sought to identify an optimal method to quantify their temporal and spatial distribution. Initial observations revealed that the majority of the signal intensity within the parenchyma originated from the lateral ventricle immediately following injection (Figure 3A). We adapted a method originally developed by Rall et al. for studying tracer diffusion following intraventricular infusion.^[^ ^6^ ^]^ To systematically analyze contrast agent distribution at different depths into the parenchyma, we delineated contiguous ROIs spanning from the lateral ventricle boundary to the cortical surface. These ROIs were categorized into four zones: Inner Medial, Inner Lateral, Outer Medial, and Outer Lateral.

**Figure 4:**
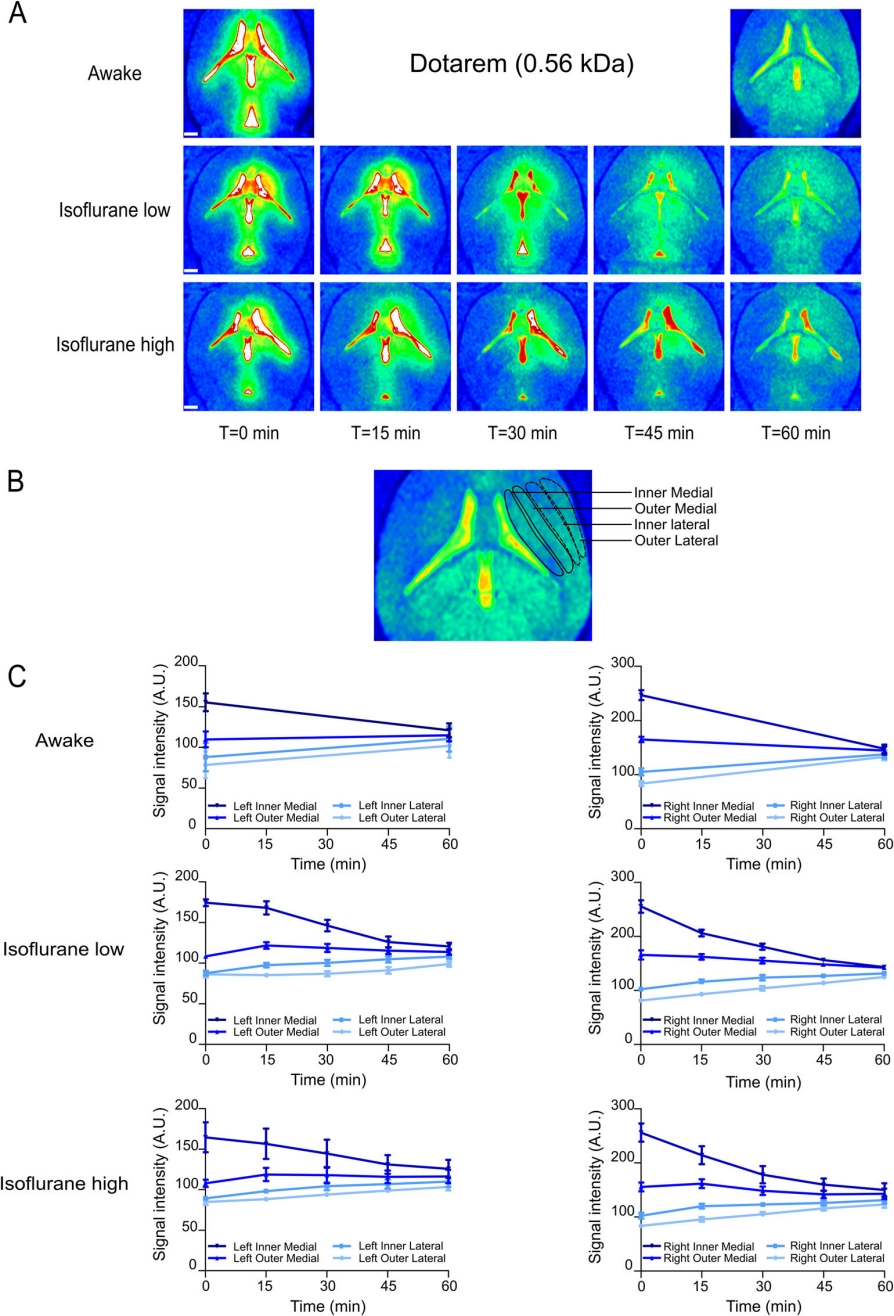
Dotarem (0.56 kDa) distribution following ventricular injection exhibits a lateral spread over time suggesting diffusion through the parenchyma A) Representative T1‐weighted MRI axial sections showing the spatiotemporal distribution of Dotarem (25 mm Gd) following a 2.5 µL intraventricular injection at 0.5 µL per min under different physiological conditions. The time‐lapse series captures the gradual lateral spread of the low molecular weight contrast agent, reaching equilibrium within 60 min. Scale bars: 1 mm. B) To quantify the lateral diffusion of Dotarem, multiple contiguous regions of interest were systematically delineated from the lateral ventricle boundary to the outer edge of the parenchyma. These ROIs were classified into four distinct zones: Inner Medial, Outer Medial, Inner Lateral, and Outer Lateral, as shown in the image. C) Quantification of signal intensity across different parenchymal regions: Inner Medial, Outer Medial, Inner Lateral, and Outer Lateral, for both the left (left column) and right (right column) hemispheres under different physiological states. Each line represents the mean signal intensity over time, based on data from *n* = 6 mice per condition, with error bars indicating the standard error of the mean (SEM). Darker lines represent inner medial regions, while lighter lines correspond to outer lateral regions, with consistent color coding applied across all conditions.

We initially tracked the diffusion of Dotarem post‐injection in axial sections using the NIH Color Look‐Up Table (CLUT) feature in the Horos software. This tool enhanced the visibility and differentiation of the contrast agent, improving image visualization and interpretability. The analysis was conducted under three distinct physiological conditions: awake, low‐dose isoflurane, and high‐dose isoflurane. Representative images in **Figure** **4**A show a spread of the tracer from the boundary of the lateral ventricle to the external cortex across all examined conditions.

The ROIs used for detailed quantification are shown in Figure 4B. Quantification of signal intensity across these ROIs revealed consistent patterns across all experimental conditions and hemispheres (Figure 4C). Specifically, the signal intensity was highest in the ROI directly adjacent to the lateral ventricle following injection and lowest in the ROIs farther from the ventricle. By 60 min post‐injection, signal intensities across all ROIs converged toward equilibrium, reflecting a concentration gradient‐driven spreading of the small contrast agent throughout the ECS of the tissue that was not affected by physiological state.

**Figure 5:**
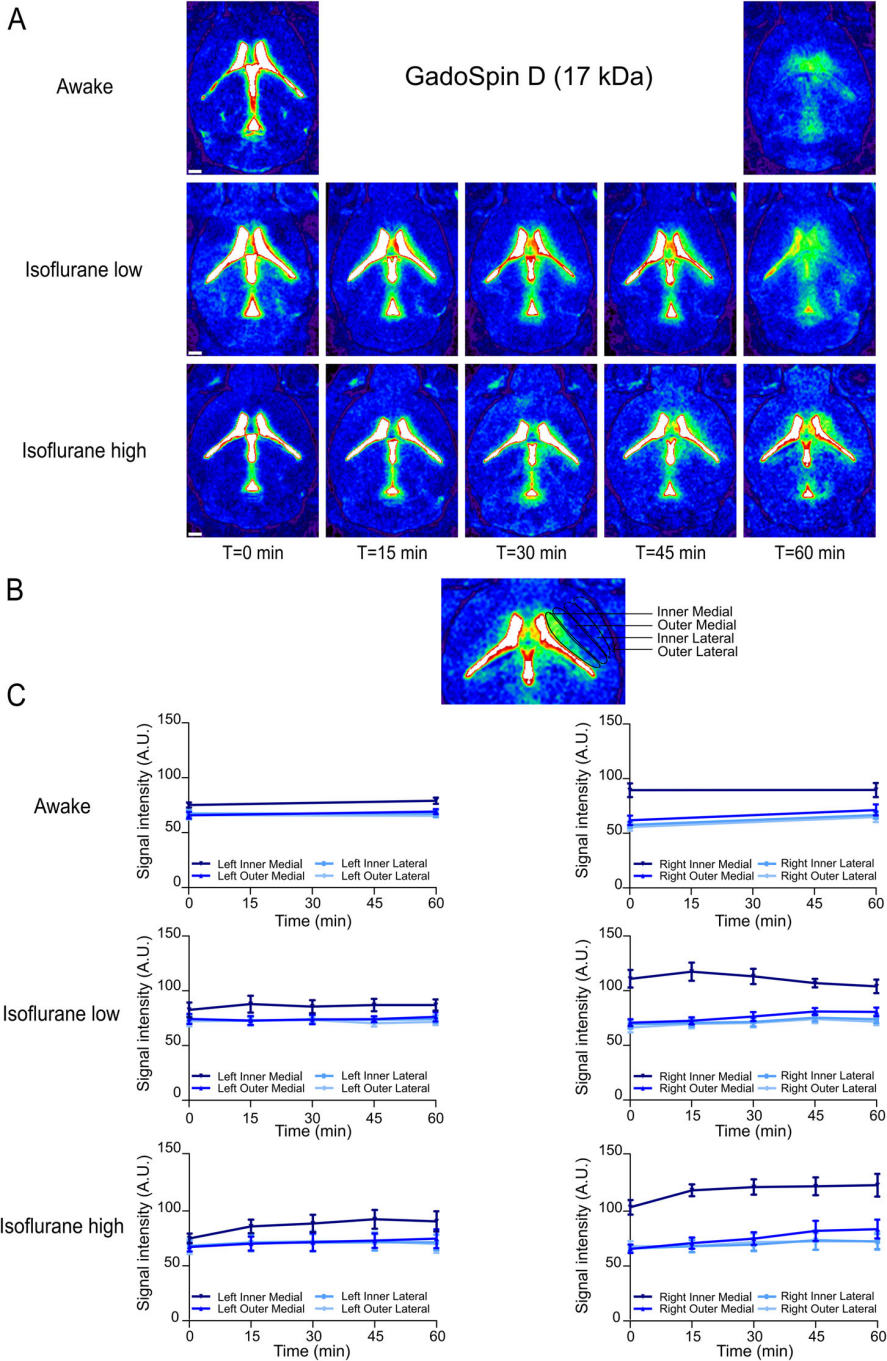
GadoSpin D (17 kDa) distribution following ventricular injection shows minimal spread through the parenchyma A) Representative T1‐weighted MRI axial sections showing the spatiotemporal distribution of GadoSpin D (25 mm Gd) following a 2.5 µL intraventricular injection at 0.5 µL per min under different physiological conditions. The time‐lapse series captures the minimal gradual lateral spread of the medium molecular weight contrast agent. The minimal spread of the tracer of mid‐size particles is consistent with diffusion‐driven tracer transport through the parenchyma. Scale bars: 1 mm. B) To quantify the lateral diffusion of GadoSpin D, multiple contiguous regions of interest were systematically delineated from the lateral ventricle boundary to the outer edge of the parenchyma. These ROIs were classified into four distinct zones: Inner Medial, Outer Medial, Inner Lateral, and Outer Lateral, as shown in the image. C) Quantification of signal intensity across different parenchymal regions: Inner Medial, Outer Medial, Inner Lateral, and Outer Lateral, for both the left (left column) and right (right column) hemispheres under different physiological states. Each line represents the mean signal intensity over time, based on data from *n =* 6 mice per condition, with error bars indicating the standard error of the mean (SEM). Darker lines represent inner medial regions, while lighter lines correspond to outer lateral regions, with consistent color coding applied across all conditions.

To evaluate whether convection, as proposed by the glymphatic hypothesis,^[^ ^26^ ^]^ also contributes to the transport of particles in the ECS, we analyzed the movement patterns of GadoSpin D, a contrast agent with a higher molecular weight. If convection plays the dominant role, the distribution patterns for molecules of varying sizes would not be expected to differ within the parenchyma. We analyzed CE‐MRI data from mice injected with GadoSpin D using the same methodology applied to Dotarem. Representative axial sections (**Figure** **5**A) revealed a minimal distribution of GadoSpin D to the parenchyma immediately following injection and a limited distribution to deeper regions at 60 min post‐injection. Using the same ROI placements as in the Dotarem analysis (Figure 5B), signal intensity measurements showed no evidence of convergence in the ROIs after 60 min (Figure 5C). This pattern was consistent across all physiological conditions and both hemispheres examined. We observed an initial increase in signal intensity within the infused ventricle at the first timepoint. However, subsequent diffusion into other regions of the parenchyma was notably limited. The limited movement of the larger particles suggests that diffusion, rather than convection, is the primary mechanism driving particle transport through the parenchymal ECS.

### 2.3 Enhanced Diffusion of Dotarem into the Olfactory Bulb Following Intraventricular Injection Under Awake and Low‐Dose‐Isoflurane Conditions

**Figure 6:**
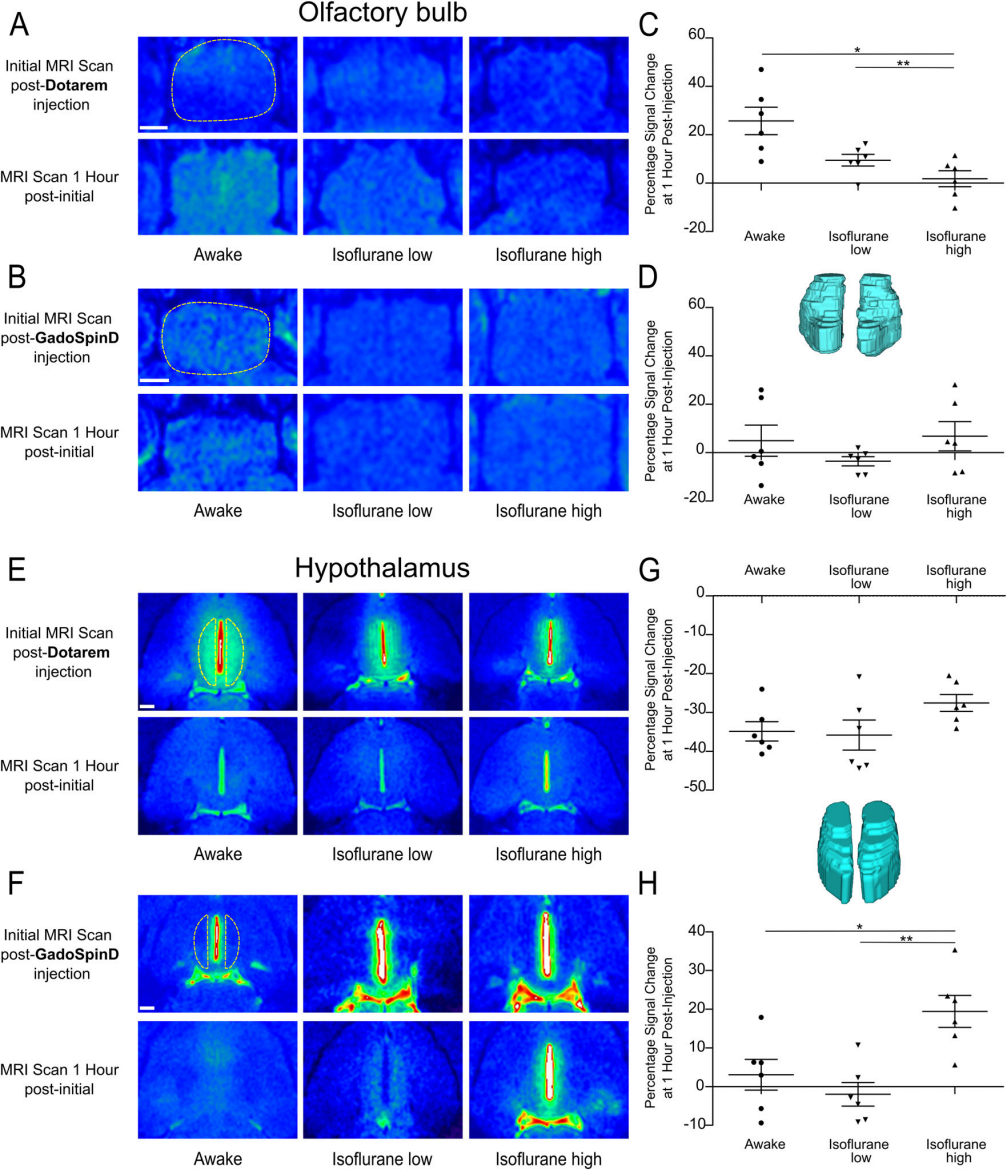
Enhanced diffusion of Dotarem (0.56 kDa) in the olfactory bulb under awake and low‐isoflurane conditions, and GadoSpin D (17 kDa) in the hypothalamus under high‐isoflurane conditions following intraventricular injection. Representative T1‐weighted MRI axial sections showing the olfactory bulb distribution of contrast agents A) Dotarem and B) GadoSpin D, both at 25 mm Gd, following intraventricular injection (2.5 µL at 0.5 µL per min). Scale bars = 0.5 mm. Regions of interest were delineated in 2D axial sections across the olfactory bulbs, followed by 3D reconstruction using Horos software to evaluate signal intensity throughout the structure. A representative 3D reconstruction of the olfactory bulbs is displayed between panels (C) and (D). C) Post‐Dotarem injection, a significant increase in signal intensity is observed in the olfactory bulbs within the first hour under awake and isoflurane low‐dose conditions compared to the high‐dose isoflurane condition. D) In contrast, GadoSpin D ventricular injection results in minimal changes in olfactory bulb signal intensity across all physiological conditions. Representative T1‐weighted MRI axial sections showing the distribution of contrast agents E) Dotarem and F) GadoSpin D, both at 25 mm Gd, following intraventricular injection (2.5 µL at 0.5 µL per min). Scale bars = 1 mm. Regions of interest were delineated in 2D axial sections across the left and right hemispheres of the hypothalamus, followed by 3D reconstruction using Horos software (version 4.0.0) to evaluate signal intensity throughout the structure. A representative 3D reconstruction of the hypothalamus is displayed between panels (G) and (H). G) Percent change in signal intensity post‐Dotarem injection demonstrates a marked reduction in the hypothalamus within the first hour across all examined physiological conditions. H) In contrast, following Gadospin D ventricular injection, minimal changes in signal intensity are observed after 1 hour under awake and low‐dose isoflurane conditions, while a significant increase occurs under high isoflurane concentrations. Data represent *n =* 6 mice. Results are presented as mean ± standard error of the mean. *p < 0.05; **p < 0.01 (one‐way ANOVA followed by Tukey's post hoc test).

Given our observation of low molecular weight contrast agent diffusion within the parenchyma localized directly adjacent to the lateral ventricles, we hypothesized that other brain regions might exhibit diffusion patterns following intraventricular injection. To investigate this, we focused on the olfactory bulb and hypothalamus, two regions indicating enhanced signal intensity, as shown in Figures 2D and 3A. False‐color mapping via NIH CLUT in Horos software revealed increased signal intensity in the olfactory bulb following intraventricular injection of Dotarem under awake and low‐dose isoflurane conditions, with no such diffusion observed for GadoSpin D (**Figure** **6**A,B). To account for the structural complexity of the olfactory bulb, ROIs were drawn on axial sections, and changes in signal intensity were analyzed using 3D models. Quantitative analysis confirmed significant diffusion of Dotarem across the whole olfactory bulb under awake and low‐dose isoflurane states compared to high‐dose isoflurane conditions (Figure 6C,D).

### 2.4 Increased Diffusion of GadoSpin D in the Hypothalamus During High‐Dose Isoflurane Conditions

Analysis of axial MRI scans demonstrated that the signal intensity of Dotarem in the hypothalamus had diminished 1 h post‐injection, regardless of the physiological state, as visualized using the NIH CLUT feature in Horos software (Figure 6E). By contrast, GadoSpin D diffusion exhibited a distinctive pattern. Under high‐dose isoflurane anesthesia, strong and persistent signal intensity was observed in the third ventricle and adjacent hypothalamus, contrasting with limited signal under awake or low‐dose isoflurane conditions (Figure 6F). These observations were confirmed in quantifications from 3D reconstructions of the hypothalamus, which revealed significantly increased signal intensity one hour after GadoSpin D injection in mice subjected to deep anesthesia compared to other conditions (Figure 6G,H).

### 2.5 Low‐Rate Infusion of a 17 kDa Contrast Agent Demonstrates a Steadily Increasing Contrast Enhancement in the Hypothalamus, Indicating Diffusion Across the Ependyma

Sustained diffusion of a moderately‐sized macromolecular contrast agent like GadoSpin D in the hypothalamus under deep anesthesia suggests distribution from the ventricles through the ependymal wall. However, considering that the injection rate of 0.5 µL per min exceeds the physiological CSF production rate in mice, we were concerned that this elevated rate may artificially promote the transependymal passage of larger particles. To address this, we designed a follow‐up experiment using a previously established infusion protocol^[^ ^27^ ^]^ that delivers contrast agents at a rate much lower than published CSF production rates for mice.^[^ ^28^, ^29^ ^]^

**Figure 7:**
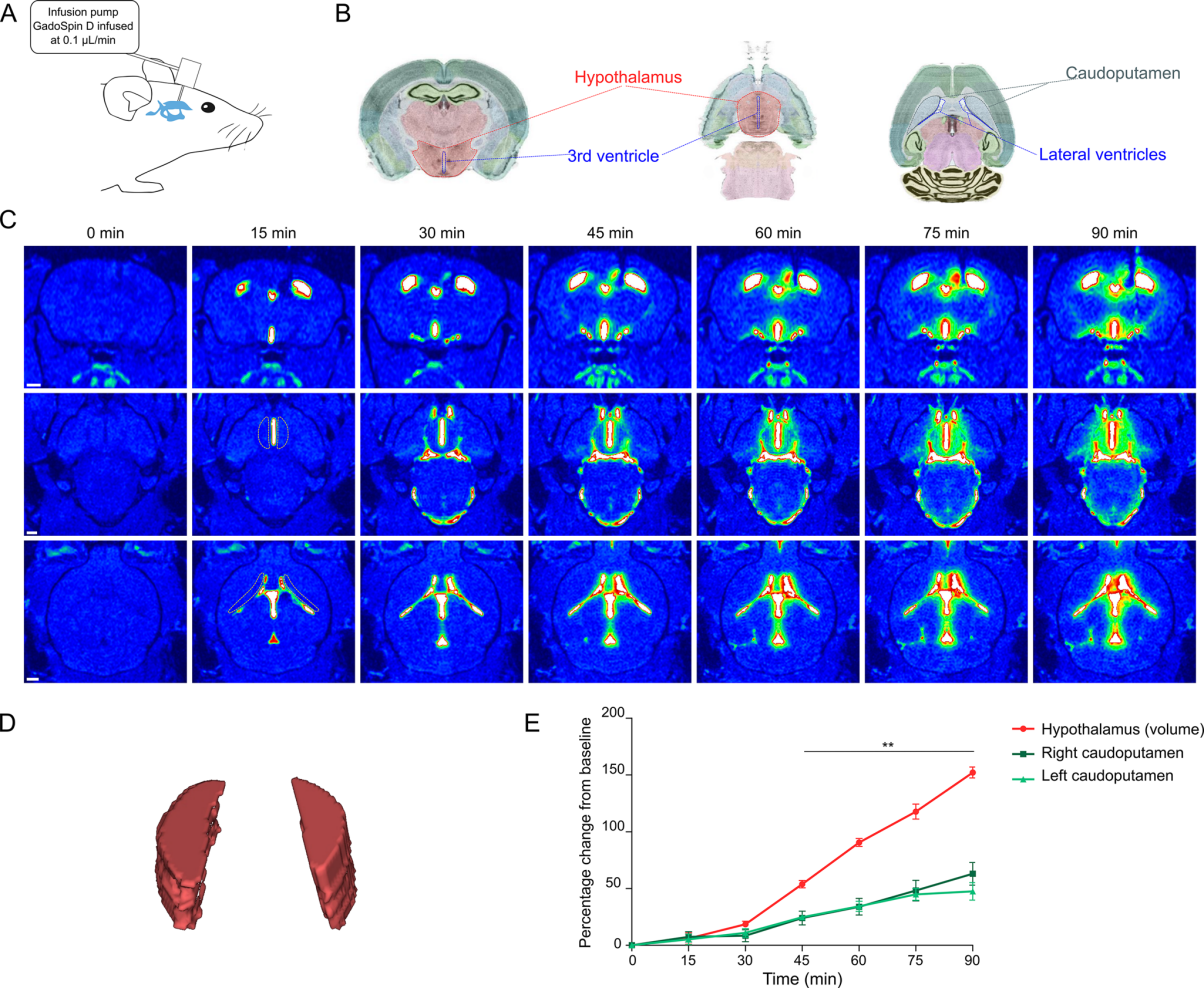
Low‐rate infusion of GadoSpin D confirms contrast enhancement in the hypothalamus, indicating diffusion of a 17 kDa contrast agent across the ependymal wall A) Schematic representation of the experimental setup. MRI‐compatible cannulae were stereotactically implanted into the right lateral ventricle of C57BL/6N mice. Following implantation, the animals were transferred to a horizontal‐bore 9.4 T MRI scanner for imaging. Polyethylene tubing, pre‐filled with GadoSpin D solution (25 mm Gd, nanoPET Pharma GmbH), connected the implanted cannula to an infusion pump. The contrast agent was delivered at a continuous rate of 0.1 µL per min. B) Anatomical annotations of the hypothalamus, third ventricle, lateral ventricles and caudoputamen. Coronal (left) and axial (middle and right) views are shown, as referenced from the Allen Mouse Brain Atlas (mouse.brain‐map.org). C) Representative T1‐weighted MRI coronal (top row), axial sections (middle and bottom) showing the spatiotemporal distribution of GadoSpin D following an intraventricular infusion at 0.1 µL per min over 90 min. The time‐lapse series depicts the gradual lateral spread of the contrast agent from the lateral ventricles into the adjacent caudoputamen, as well as from the third ventricle into the hypothalamus. Regions of interest were delineated in 2D axial sections surrounding the hypothalamus and caudoputamen in both the left and right hemispheres. To assess signal intensity throughout the hypothalamus structure, a 3D reconstruction D) was generated using Horos software (version 4.0.0). E) Signal dynamics of GadoSpin D tracer efflux into the hypothalamus and the right and left caudoputamen. Data are expressed as mean ± SEM, *n =* 6 mice. **p < 0.01, determined by 2‐way ANOVA followed by Bonferroni's post hoc test. Scale bars: 1 mm.

As illustrated in **Figure** **7**A, an MRI‐compatible cannula was stereotactically implanted into the lateral ventricle and connected to an infusion pump for controlled delivery at low infusion rates. While the mouse was positioned in the magnet, GadoSpin D was infused at a continuous rate of 0.1 µL per min. ROIs were delineated in 2D coronal and axial MRI sections, with a specific focus on the hypothalamus and caudoputamen (Figure 7B). Temporal imaging revealed the progressive diffusion of GadoSpin D over 90 min, with pronounced contrast enhancement in both the hypothalamic and caudoputamen regions (Figure 7C). We further observed that diffusion was not more pronounced in the infused ventricle compared to the contralateral ventricle, suggesting that the low‐rate infusion mitigated transependymal contrast enhancement caused by the injection. To better assess the structural complexity of the hypothalamus, detailed 3D reconstructions were performed (Figure 7D). Quantifications of the contrast agent confirmed an increase over time in the diffusion of GadoSpin D through the caudoputamen and the hypothalamus. Notably, signal intensity quantification revealed significantly greater accumulation of the 17 kDa contrast agent in hypothalamic regions adjacent to the third ventricle compared to the caudoputamen adjacent to the lateral ventricle as early as 45 min. These findings suggest that the ependyma, particularly lining the third ventricle, is permeable to moderately sized contrast agents allowing their spread into the parenchyma after ventricular administration.

## 3 Discussion and Conclusions

**Figure 8:**
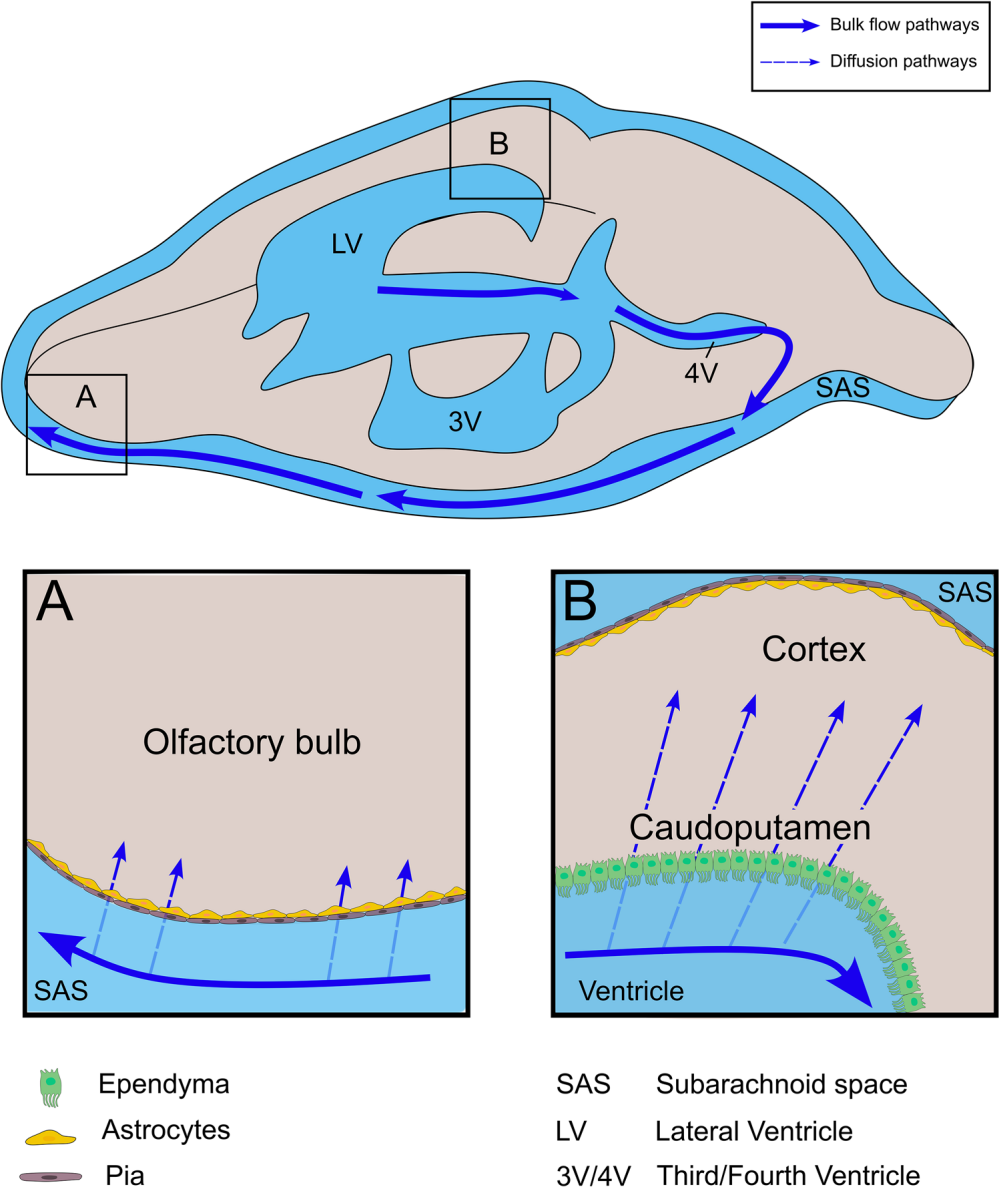
Summary of bulk CSF flow pathways and interfaces for the diffusion of contrast agents between the CSF and brain parenchyma. A schematic overview of the mouse brain and CSF‐containing ventricular (LV, 3V, and 4V) and subarachnoid spaces (SAS) is shown. After LV administration, contrast agents clear by convective bulk flow (solid blue arrows) from the ventricular spaces to reach the SAS on the ventral aspect of the brain, eventually clearing along efflux pathways that include routes along olfactory nerve bundles to lymphatic vessels (not shown). During awake or low‐dose isoflurane anesthesia conditions, bulk clearance out of the ventricles was more evident leading to increased distribution of contrast agents toward the olfactory bulbs. Under these conditions, an increased presence of the low‐molecular‐weight contrast agent was measured in the olfactory bulb, which we propose occurred by diffusion (dashed blue arrows) across the pia‐glia interface into the parenchyma as shown in box A. During high‐dose isoflurane conditions, bulk clearance out of the ventricles was inhibited. Under these impaired clearance conditions, an increase in signal intensity of the 17 kDa contrast agent was detected in periventricular brain regions. We propose, as seen in box B, that diffusion of contrast agents occurs directly from the ventricular CSF to the brain parenchyma through the semi‐permeable ependyma. Once within the parenchyma, low molecular weight contrast agents distribute rapidly throughout the extracellular space of the brain while the spread of higher molecular weight contrast agents is a much slower process, consistent with diffusion. The potential distribution of contrast agents from the SAS to the brain along penetrating perivascular spaces (e.g. at the dorsal cortex) could not be assessed in the current study.

In this study, we employed two different molecular weight (0.56 and 17 kDa) gadolinium‐based tracers in combination with DCE‐MRI to explore CSF circulation in mice across different physiological states. Our results demonstrate that CSF clearance from the lateral ventricles was markedly increased in awake mice and under low‐dose isoflurane anesthesia compared to high‐dose isoflurane anesthesia. Monitoring of signal intensity within the brain parenchyma over time revealed that the 17 kDa molecular weight contrast agent exhibited a significantly reduced spread compared to the low molecular weight contrast agent, supporting diffusion as the primary transport mechanism in the parenchymal ECS. Notably, physiological state‐dependent modulation of diffusion was observed in specific brain regions as the low molecular weight contrast agent displayed enhanced distribution to the olfactory bulb under awake and low‐dose anesthesia conditions, whereas the larger contrast agent only showed diffusion into the hypothalamus under high‐dose anesthesia. Finally, employing a low‐rate infusion method, we established that diffusion of contrast agent through the ependymal wall reflects a physiological‐relevant process rather than being an artifact induced by injection into the ventricle. The proposed roles of bulk flow and diffusion in the distribution of contrast agents to the brain have been summarized in schematic form in **Figure** **8**.

The mechanisms of solute movement between the brain ISF and CSF are currently intensely debated.^[^ ^30^, ^31^ ^]^ The dominant model in the field, the glymphatic model, which proposes a convective flow of CSF and solutes into and through the brain parenchyma,^[^ ^13^ ^]^ runs counter to a wealth of historical evidence that has highlighted diffusion as the predominant physiological mechanism for solute movement.^[^ ^30^ ^]^ Much of this historical work examined the distribution of radiolabeled solutes after injections into the ventricles or cisterna magna.^[^ ^32^, ^33^ ^]^ After sacrifice at various time points after the injections, the solutes demonstrated a pattern consistent with diffusion from the CSF compartment to the parenchyma, through interfaces at the pia‐glia or the ependymal wall of the brain ventricle.^[^ ^6^, ^33^, ^34^ ^]^ Electron microscopy studies utilizing electron‐dense tracers such as horseradish peroxidase indicated that the cells lining the interfaces between CSF and the parenchyma do not form a barrier.^[^ ^35^ ^]^ This conclusion was further supported by in vivo imaging using low‐resolution MRI and a small contrast agent, where Bui et al. observed diffusion of the tracer from the lateral ventricle into the surrounding parenchyma.^[^ ^36^ ^]^ Quantitative studies from tissue sections of the spread of different‐sized tracers into the brain from the ventricles have provided data that fit models of simple diffusion, albeit at rate constants much lower than what would be expected in aqueous solutions due to the tortuosity of the extracellular space.^[^ ^32^ ^]^ A model of a ‘CSF sink’ was devised, where bulk flow in the CSF compartment helps determine the direction of solute movement based on local concentration differences at the interfaces between the CSF and parenchyma.^[^ ^12^ ^]^ In this model, the bulk turnover of CSF would help drive clearance for metabolites from the brain parenchyma, while at the same time allowing passage of certain hormones or nutrients from the CSF to the brain.

Our study was designed to test the CSF sink model in vivo by evaluation using DCE‐MRI of the spread of two different‐sized contrast agents to and within the parenchyma during conditions in which the bulk clearance from the ventricles was expected to vary. We adapted a method originally developed by Rall et al.^[^ ^6^ ^]^ for studying tracer diffusion following intraventricular injection in live dogs. In their study, animals were infused for 3–5 h with radiolabeled inulin, after which the brains were harvested and sectioned coronally. From these sections, smaller adjacent subregions extending from the lateral ventricle boundary to the cortical surface were dissected, and the radioactivity in each subregion was quantified to assess tracer propagation. In our study, we employed contrast‐enhanced MRI (CE‐MRI) to noninvasively monitor tracer propagation over time in living animals. In contrast to Rall et al., who relied on postmortem physical brain sectioning, our approach utilized virtual sectioning in vivo, preserving as much of the individual physiological conditions as possible.

Our data fit a pattern that would be expected by the CSF sink model, with diffusion as the dominant mechanism of exchange between the CSF compartment and the parenchyma. At the same time, our results call into question the model of the glymphatic system in which CSF and solutes were shown to mostly enter the brain along perivascular spaces from the SAS, rather than by diffusion through the ependymal wall.^[^ ^13^ ^]^ Importantly, perivascular entry from the ventricular space to the brain parenchyma would not be anatomically feasible, as blood vessels do not directly enter the brain through the ependyma.

By monitoring the clearance over time from the ventricles as a measure of CSF turnover, our results also challenge the popular notion that CSF flow is reduced during waking conditions and enhanced during deep anesthesia or sleep.^[^ ^14^ ^]^ In their pioneering work, Xie et al. employed sleep as a natural physiological state to conduct their experiments.^[^ ^14^ ^]^ However, replicating this approach was technically infeasible in our study due to two primary limitations. First, inducing sleep in mice immediately following intraventricular injection of the contrast agent was not achievable within our experimental framework. Second, the high acoustic noise generated by the MRI scanner rendered it impossible to sustain a sleep state in the animals, unlike the controlled conditions utilized by Xie et al. with two‐photon imaging. We adopted anesthesia as the most viable alternative. Isoflurane was selected over ketamine due to its distinct advantages. Isoflurane allows for precise control of anesthesia depth and enables rapid recovery, facilitating the immediate awakening of the animals post‐intraventricular injection, an outcome not attainable with ketamine. Moreover, to ensure methodological consistency, all animals were subjected to isoflurane anesthesia during both the initial phase of the experiment and the MRI scanning sessions. This uniform approach reduced variability and preserved the integrity of our experimental design.

Using this experimental paradigm, we demonstrated that CSF clearance from the ventricular system was significantly influenced by the physiological state and was independent of the molecular weight of the contrast agents used. By employing two paramagnetic tracers of different molecular weights, we found that CSF clearance was markedly enhanced in mice that were awake or under low‐dose anesthesia compared to high‐dose anesthesia. This was evident from the reduced retention of contrast agents within the ventricular system and adjacent to the circle of Willis 1 h post‐injection. These findings align with our previous near‐infrared imaging studies, which showed that 40 kDa PEG‐conjugated tracer exhibited significantly higher CSF outflow to the periphery in awake mice than in anesthetized ones.^[^ ^17^ ^]^ In their initial study, Xie et al.^[^ ^14^ ^]^ reported elevated tracer signals in the subarachnoid space during sleep and anesthetic states compared to the awake condition but did not consider peripheral tracer clearance. Although they concluded that sleep drives CSF clearance, an alternative interpretation could be that the increased signal intensity in the subarachnoid space and adjacent brain parenchyma reflects reduced CSF outflow to peripheral compartments rather than enhanced tracer influx into the brain parenchyma. This hypothesis aligns with our prior findings, where postmortem analyses revealed an inverse correlation between tracer accumulation along perivascular spaces on the surface of the brain and CSF outflow rates.^[^ ^17^ ^]^

Consistently, our current data show that a higher dose of isoflurane anesthesia significantly impaired tracer clearance from the ventricular system. One potential explanation for this effect is that isoflurane is known to dilate cerebral blood vessels in a dose‐dependent manner, including pial arteries.^[^ ^37^, ^38^, ^39^ ^]^ Thus, under high‐dose isoflurane, this may reduce CSF flow toward the basal cisterns and major efflux pathways in mice by increasing the flow resistance in the SAS, as proposed in a previous MRI study.^[^ ^40^ ^]^ Simultaneously, under these reduced clearance conditions, we observed a marked increase in the diffusion of the 17 kDa tracer GadoSpin D from the third ventricle to the adjacent hypothalamus via the ependymal layer. Given the absence of penetrating arteries in this region, we concluded that impaired ventricular tracer clearance establishes a concentration gradient across the ependymal wall, promoting the diffusion of mid‐sized molecules into the surrounding parenchyma. It is important to note that in their study Xie et al. focused primarily on arterial perivascular spaces as inflow pathways and did not acknowledge diffusion driven by concentration gradients between the CSF and parenchyma as a mechanism for tracer distribution. It should be acknowledged that from the current data, we cannot rule out that an entry of contrast agents from the SAS to the brain parenchyma may also occur along penetrating perivascular spaces.

The notion that the conditions of sleep or deep anesthesia actively promote the clearance of metabolites from the brain has been challenged by others. Recently, Miao et al.^[^ ^19^ ^]^ have concluded that the clearance of fluorescent molecules from the mouse brain was, in fact, significantly reduced during sleep and anesthetic states. In addition, Gakuba et al.^[^ ^18^ ^]^ conducted an MRI study reporting enhanced contrast agent influx into the brain during awake conditions. A common limitation of these two studies is that their experimental designs did not allow assessment of the initial tracer dose to adequately account for the inter‐animal variability inherent in administering small volumes in vivo. In our study, by administering contrast agents into the lateral ventricle, we were able to perform baseline scans shortly after the infusions. When using the same contrast agent as employed by Gakuba et al., we similarly observed that ventricular infusion of Dotarem led to increased tracer accumulation in the olfactory bulb during both awake conditions and low‐dose isoflurane anesthesia. One potential explanation is that under awake conditions a greater concentration of tracer may reach the basal cisterns, allowing small molecules to diffuse into the adjacent parenchyma. This aligns with previous observations of small contrast agents diffusing from the ventral SAS to the parenchyma, as described in earlier studies, including initial MRI‐based investigations of the glymphatic system.^[^ ^20^ ^]^ When employing a larger contrast agent, GadoSpin D, no significant accumulation in the olfactory bulb over time was observed during any of the physiological states analyzed. This finding supports the hypothesis that moderately sized molecules face greater diffusion restrictions from the SAS to the parenchyma across the pia‐glia interface.

Despite structural differences, both Dotarem and GadoSpin D share highly similar chelating structures, with comparable charge and hydrophilicity. Dotarem (Gd‐DOTA) is a small‐molecule contrast agent based on a cyclic Gd‐chelate, while GadoSpin D is a macromolecular contrast agent—a dendrimer—functionalized with multiple cyclic Gd‐chelates. Thus, we conclude that the primary distinguishing factor between these agents is their molecular size. Although we cannot exclude the potential influence of other intrinsic molecular properties—such as shape, electrostatic interactions, size (radius of gyration), conformation, or other physical/chemical interactions— our interpretation aligns with that of the Benveniste group, who attributed observed differences in CSF‐ISF exchange patterns between <1 kDa Gd‐DTPA tracer and  200 kDa GadoSpin P to molecular size differences.^[^ ^20^ ^]^ Our results align with our previous work utilizing bolus infusions of 40‐kDa PEG‐conjugated tracers and NIR imaging, which similarly revealed no obvious diffusion on post‐mortem sections.

In this study, we also observed that the injection of Dotarem or GadoSpin D into the ventricles resulted in the immediate detection of the contrast agent within deep brain regions, such as the caudoputamen, located adjacent to the lateral ventricles. Low‐rate infusion experiments further confirmed that the diffusion of medium‐sized molecules from the ventricles to the parenchyma is a physiological‐relevant process and is not solely attributable to injection pressure. Remarkably, GadoSpin D exhibited significantly enhanced diffusion within the subependymal zone of the third ventricle compared to the lateral ventricle. This substantial diffusion of moderately sized molecules from the third ventricle may have profound physiological implications, potentially facilitating access to the hypothalamus for critical regulatory molecules, such as hormones and neuropeptides, that are essential for maintaining homeostasis. Further work should explore how anatomical differences between different brain regions might influence the diffusion of molecules.

In summary, our findings using dynamic in vivo MRI have provided evidence for a concentration‐dependent diffusion of contrast agents to the parenchyma across the ependyma, a conclusion consistent with previous observations.^[^ ^6^, ^33^, ^34^ ^]^ Our data has also highlighted the importance of the role of the rate of bulk clearance from the ventricles in this process. These results shed light on the mechanisms of solute exchange between the CSF and the brain parenchyma and may inform future approaches for drug delivery to the CNS.

## 4 Experimental Section

### Mice

Wild‐type male and female mice (C57BL/6N background), obtained either from Janvier Labs (Le Genest‐Saint‐Isle, France) or bred in‐house, were maintained under specific pathogen‐free conditions. Mice were kept in individually ventilated cages at a light‐dark cycle of 13 h–11 h. Food and water were accessible ad libitum. All mice were used for experiments at 2 to 4 months of age. The distribution of males and females was balanced between the groups.

### Intraventricular Contrast Agent Injection

Mice were first anesthetized with 3% isoflurane using an induction chamber, followed by subcutaneous administration of carprofen for analgesia. The animals were then positioned in a stereotaxic frame (Kopf Instruments, Tujunga, CA, USA). Under 2–3 % isoflurane anesthesia, the skull was thinned using an engraving drill. A 33‐gauge steel needle was stereotactically inserted into the right lateral ventricle at coordinates 0.95 mm lateral, 0.22 mm caudal relative to the bregma, and 2.4 mm ventral from the skull surface. Contrast agents, either Dotarem (25 mm Gd, Guerbet, Paris, France) or GadoSpin D solution (25 mm Gd, nanoPET Pharma GmbH, Germany), were injected for 5 min using a NanoJet syringe pump (Chemyx, Stafford, CT, USA) at a controlled rate of 0.5 µL per min. Following the injection, the needle was left in place for an additional 5 min to ensure uniform distribution of the contrast agent throughout the ventricle. Following the intraventricular injection, the mice were placed in a prone position on an MRI cradle (BioSpec Avance III 94/20, Bruker BioSpin GmbH, Ettlingen, Germany) for an initial MRI scan. After this scan, the animals were either maintained under isoflurane anesthesia at low concentrations (1–1.5%) or high concentrations (2–3%) or allowed to recover in individual cages for 5–10 min and then remained awake for the remainder of a 60 min period (including recovery time) before the final imaging session. Animals under isoflurane anesthesia were imaged at 15 min intervals, with the final scan taking place 60 min after the initial scan.

Mice exposed to a high dose of isoflurane had a breathing rate of 20–30 breaths per minute, whereas those under a low dose exhibited a significantly higher rate of 110–150 breaths per minute. Mice breathed spontaneously during the procedure, and respiratory rate and body temperature (36.5–37.5 °C) were continuously monitored using noninvasive probes (SA Instruments, Stony Brook, NY, USA).

### Low‐Rate Intraventricular Contrast Agent Infusion

Low‐rate infusions into the lateral ventricle were performed as previously described.^[^ ^27^ ^]^ Briefly, mice were anesthetized via intraperitoneal injection of ketamine (100 mg kg^−1^) and xylazine (20 mg kg^−1^) and securely positioned in a stereotaxic frame (Kopf Instruments). Under anesthesia, the skull was thinned using an engraving drill. A 28‐gauge, 2.5 mm MRI‐compatible microcannula (328OP/PK/Spc; Plastics One, Bilaney Consultants, Düsseldorf, Germany) was stereotactically inserted at coordinates 0.95 mm lateral, 0.22 mm caudal to the bregma, and 2.50 mm ventral from the skull surface. The cannula was sealed using cyanoacrylate glue. The animals were then transferred in a prone position onto an MRI cradle (BioSpec Avance III 94/20; Bruker BioSpin GmbH) for imaging. A polyethylene catheter (1 to 1.5 m) filled with a GadoSpin D solution (25 mm Gd; nanoPET Pharma GmbH) was connected to the microcannula and a 10 µL syringe operated by an MRI‐compatible NanoJet syringe pump (Chemyx Inc.). The surgical site was closed with a medical adhesive bandage surrounding the cannula and catheter. Mice were allowed to breathe spontaneously throughout the entire procedure, with respiratory rate and body temperature continuously monitored using noninvasive probes (SA Instruments). Core body temperature was maintained between 36.5 and 37.5 °C. During MRI acquisition, anesthesia was supplemented as needed with 0.5%–1% isoflurane, ensuring a respiratory rate below 140 breaths per min. The contrast agent was infused at a constant rate of 0.1 µL per min for 90 min.

### Magnetic Resonance Imaging

MRI was performed using a horizontal‐bore 9.4 T animal scanner (BioSpec Avance III 94/20; Bruker BioSpin GmbH) equipped with a BGA12S gradient system and operated with ParaVision 6.0.1 (Bruker BioSpin GmbH). A linearly polarized coil with an inner diameter of 40 mm (Bruker BioSpin GmbH) was used for signal reception. Contrast‐enhanced imaging was achieved using a 3D time‐of‐flight gradient recalled echo sequence, originally optimized for imaging peripheral lymphatic vessels.^[^ ^41^ ^]^ Imaging parameters included a recovery time of 12.0 ms, echo time of 2.5 ms, flip angle of 25°, matrix size of 600 × 432 × 180, field of view of 36.00 mm × 25.92 mm × 18.00 mm, 1 signal average, and a total acquisition time of 4 min, 19.2 s. A calibration phantom (5 mm Gd in 0.9% NaCl) positioned near the animal's head was used for image intensity normalization across the time series.

### Data Processing

Data were processed using a method adapted from that previously described by Iliff et al.^[^ ^20^ ^]^ The MRI processing workflow comprised head motion correction, intensity normalization, and spatial smoothing. First, the T1‐weighted MRI images were exported in DICOM format and then converted to the NIfTI format with the MRIcron software (https://www.nitrc.org/projects/mricron). SPM12 (http://www.fil.ion.ucl.ac.uk/spm/) was next employed for image processing. Scan‐to‐scan misregistration due to head movement was corrected using rigid‐body alignment for each individual scan. The image intensity was normalized throughout the time series by dividing each voxel's intensity by the mean intensity of the reference phantom placed near the animal's head. The reference phantom signal intensity value was determined using MRIcron software, where a 3D region of interest was delineated around the phantom, and the mean signal intensity within this ROI was extracted. The resulting values were then multiplied by a factor of 1000. Finally, isotropic Gaussian smoothing with a full width at half maximum of 0.1 mm was applied voxel‐wise to the image intensity.

The processed scans were then imported into the software Horos (version 3.3.6, Horos Project). ROIs were manually drawn around the different anatomical regions investigated. 3D reconstructions were performed when stated. Contrast agent distribution over time was determined by calculating the percentage change of signal intensity as a function of time after the initial scan using the following equation:

(1) $$\begin{array}{ccc} & & \left[\left(\mathrm{normalized}\;\mathrm{signal}\;\mathrm{intensity}-\mathrm{normalized}\;\mathrm{initial}\;\mathrm{scan}\;\mathrm{intensity}\right)\right.\\ & & \left.\left(\mathrm{normalized}\;\mathrm{initial}\;\mathrm{scan}\;\mathrm{intensity}\right)\right]\times 100 \end{array}$$

### Statistics

All statistical analyses were conducted using GraphPad Prism version 5 (GraphPad Software, La Jolla, CA, USA). Data are presented as mean ± standard error of the mean (SEM). For group comparisons under the awake, low‐dose, or high‐dose isoflurane conditions, a one‐way analysis of variance (ANOVA) was applied, followed by Tukey's multiple comparison post hoc test to identify specific group differences. To evaluate time‐dependent changes and regional effects, a two‐way ANOVA was used, with time points treated as a within‐subject factor and ROI location as a between‐subject factor. Post hoc analyses were performed using Bonferroni's correction to account for multiple comparisons. A p‐value of <0.05 was considered to indicate statistical significance.

### Study Approval

All animal experiments were conducted in compliance with institutional and national ethical guidelines and were approved by the Landesamt für Gesundheit und Verbraucherschutz, Saarbrücken, Germany, under license numbers 31/2018 and 45/2019.

## Conflict of Interest

The authors declare no conflict of interest.

## Author Contributions

Y.D. and S.T.P. conceived and designed the study. Y.D. and A.M. performed the MRI experiments. Y.D., A.M., and S.T.P. analyzed the data. Y.D. and S.T.P. drafted the manuscript. All authors have approved the final version of the manuscript and have agreed to be accountable for all aspects of the work.

## Data Availability

The data that support the findings of this study are available from the corresponding author upon reasonable request.

## References

[1] M. L. Rennels , T. F. Gregory , O. R. Blaumanis , K. Fujimoto , P. A. Grady , Brain Res. 1985, 326, 47. 3971148 10.1016/0006-8993(85)91383-6

[2] L. H. Weed , Physiol. Rev. 1922, 2, 171.

[3] H. F. Cserr , D. N. Cooper , P. K. Suri , C. S. Patlak , Am. J. Physiol. 1981, 240, F319. 7223889 10.1152/ajprenal.1981.240.4.F319

[4] H. Cserr , Am. J. Physiol. 1965, 209, 1219. 5846924 10.1152/ajplegacy.1965.209.6.1219

[5] T. L. Roth , D. Nayak , T. Atanasijevic , A. P. Koretsky , L. L. Latour , D. B. McGavern , Nature. 2014, 505, 223. 24317693 10.1038/nature12808 PMC3930079

[6] D. P. Rall , W. W. Oppelt , C. S. Patlak , Life Sci. 1962, 1, 43.

[7] J. F. Ghersi‐Egea , W. Finnegan , J. L. Chen , J. D. Fenstermacher , Neuroscience. 1996, 75, 1271. 8938759 10.1016/0306-4522(96)00281-3

[8] I. Klatzo , J. Miquel , P. J. Ferris , J. D. Prokop , D. E. Smith , J Neuropathol Exp Neurol. 1964, 23, 18. 14105307 10.1097/00005072-196401000-00002

[9] K. E. Holter , B. Kehlet , A. Devor , T. J. Sejnowski , A. M. Dale , S. W. Omholt , O. P. Ottersen , E. A. Nagelhus , K. A. Mardal , K. H. Pettersen , Proc Natl Acad Sci U S A. 2017, 114, 9894. 28847942 10.1073/pnas.1706942114 PMC5604020

[10] C. Nicholson , S. Hrabetova , Biophys. J. 2017, 113, 2133. 28755756 10.1016/j.bpj.2017.06.052 PMC5700249

[11] B. J. Jin , A. J. Smith , A. S. Verkman , J. Gen. Physiol. 2016, 148, 489. 27836940 10.1085/jgp.201611684 PMC5129742

[12] H. Davson , in Physiology of the Cerebrospinal Fluid, J. & A. Churchill, London 1967.

[13] J. J. Iliff , M. Wang , Y. Liao , B. A. Plogg , W. Peng , G. A. Gundersen , H. Benveniste , G. E. Vates , R. Deane , S. A. Goldman , E. A. Nagelhus , M. Nedergaard , Sci. Transl. Med. 2012, 4, 147ra111. 10.1126/scitranslmed.3003748 PMC3551275 22896675

[14] L. Xie , H. Kang , Q. Xu , M. J. Chen , Y. Liao , M. Thiyagarajan , J. O'Donnell , D. J. Christensen , C. Nicholson , J. J. Iliff , T. Takano , R. Deane , M. Nedergaard , Science. 2013, 342, 373. 24136970 10.1126/science.1241224 PMC3880190

[15] M. Asgari , D. de Zelicourt , V. Kurtcuoglu , Sci. Rep. 2016, 6, 38635. 27929105 10.1038/srep38635 PMC5144134

[16] A. J. Smith , X. Yao , J. A. Dix , B. J. Jin , A. S. Verkman , Elife. 2017, 6, e27679. 28826498 10.7554/eLife.27679 PMC5578736

[17] Q. Ma , M. Ries , Y. Decker , A. Müller , C. Riner , A. Bücker , K. Fassbender , M. Detmar , S. T. Proulx , Acta Neuropathol. 2019, 137, 151. 30306266 10.1007/s00401-018-1916-x PMC6338719

[18] C. Gakuba , T. Gaberel , S. Goursaud , J. Bourges , C. Di Palma , A. Quenault , S. Martinez de Lizarrondo , D. Vivien , M. Gauberti , Theranostics. 2018, 8, 710. 29344300 10.7150/thno.19154 PMC5771087

[19] A. Miao , T. Luo , B. Hsieh , C. J. Edge , M. Gridley , R. T. C. Wong , T. G. Constandinou , W. Wisden , N. P. Franks , Nat. Neurosci. 2024, 27, 1425. 10.1038/s41593-024-01698-0 PMC11239483 38877307

[20] J. J. Iliff , H. Lee , M. Yu , T. Feng , J. Logan , M. Nedergaard , H. Benveniste , J. Clin. Invest. 2013, 123, 1299. 23434588 10.1172/JCI67677 PMC3582150

[21] R. S. Gomolka , L. M. Hablitz , H. Mestre , M. Giannetto , T. Du , N. L. Hauglund , L. Xie , W. Peng , P. M. Martinez , M. Nedergaard , Y. Mori , Elife. 2023, 12, e82232. 36757363 10.7554/eLife.82232 PMC9995113

[22] H. Lee , K. Mortensen , S. Sanggaard , P. Koch , H. Brunner , B. Quistorff , M. Nedergaard , H. Benveniste , Magn Reson Med. 2018, 79, 1568. 28627037 10.1002/mrm.26779 PMC5736474

[23] T. Gaberel , C. Gakuba , R. Goulay , S. Martinez De Lizarrondo , J. L. Hanouz , E. Emery , E. Touze , D. Vivien , M. Gauberti , Stroke. 2014, 45, 3092. 25190438 10.1161/STROKEAHA.114.006617

[24] H. Mestre , L. M. Hablitz , A. L. Xavier , W. Feng , W. Zou , T. Pu , H. Monai , G. Murlidharan , R. M. Castellanos Rivera , M. J. Simon , M. M. Pike , V. Pla , T. Du , B. T. Kress , X. Wang , B. A. Plog , A. S. Thrane , I. Lundgaard , Y. Abe , M. Yasui , J. H. Thomas , M. Xiao , H. Hirase , A. Asokan , J. J. Iliff , M. Nedergaard , Elife. 2018, 7, e40070. 30561329 10.7554/eLife.40070 PMC6307855

[25] P. K. Eide , G. Ringstad , Acta Radiol Open. 2015, 4, 2058460115609635. 26634147 10.1177/2058460115609635 PMC4652208

[26] T. Bohr , P. G. Hjorth , S. C. Holst , S. Hrabetova , V. Kiviniemi , T. Lilius , I. Lundgaard , K. A. Mardal , E. A. Martens , Y. Mori , U. V. Nagerl , C. Nicholson , A. Tannenbaum , J. H. Thomas , J. Tithof , H. Benveniste , J. J. Iliff , D. H. Kelley , M. Nedergaard , iScience 2022, 25, 104987. 36093063 10.1016/j.isci.2022.104987 PMC9460186

[27] Y. Decker , J. Kramer , L. Xin , A. Muller , A. Scheller , K. Fassbender , S. T. Proulx , JCI Insight. 2022, 7, e150881. 34905509 10.1172/jci.insight.150881 PMC8855808

[28] H. Davson , M. B. Segal , Physiology of the CSF and Blood Brain Bariers, CRC‐Press, USA 1995.

[29] A. B. Steffensen , E. K. Oernbo , A. Stoica , N. J. Gerkau , D. Barbuskaite , K. Tritsaris , C. R. Rose , N. MacAulay , Nat. Commun. 2018, 9, 2167. 29867199 10.1038/s41467-018-04677-9 PMC5986890

[30] A. J. Smith , A. S. Verkman , FASEB J. 2018, 32, 543. 29101220 10.1096/fj.201700999 PMC5888402

[31] S. B. Hladky , M. A. Barrand , Fluids Barriers CNS. 2022, 19, 9. 35115036 10.1186/s12987-021-00282-z PMC8815211

[32] J. D. Fenstermacher , C. S. Patlak , in Dynamics of brain edema, Springer, Berlin Heidelberg 1976, 87.

[33] M. G. Proescholdt , B. Hutto , L. S. Brady , M. Herkenham , Neuroscience. 2000, 95, 577. 10658638 10.1016/s0306-4522(99)00417-0

[34] J. C. Lee , J. Olszewski , Neurology. 1960, 10, 814. 13760238 10.1212/wnl.10.9.814

[35] M. W. Brightman , T. S. Reese , J. Cell Biol. 1969, 40, 648. 5765759 10.1083/jcb.40.3.648 PMC2107650

[36] J. D. Bui , D. R. Nammari , D. L. Buckley , B. A. Inglis , X. S. Silver , T. H. Mareci , M. I. Phillips , Neuroscience. 1999, 90, 1115. 10218810 10.1016/s0306-4522(98)00519-3

[37] H. Iida , H. Ohata , M. Iida , Y. Watanabe , S. Dohi , Anesthesiology. 1998, 89, 954. 9778013 10.1097/00000542-199810000-00020

[38] C. T. Sullender , L. M. Richards , F. He , L. Luan , A. K. Dunn , J. Neurosci. Methods. 2022, 366, 109434. 34863840 10.1016/j.jneumeth.2021.109434 PMC9258779

[39] B. F. Matta , K. J. Heath , K. Tipping , A. C. Summors , Anesthesiology. 1999, 91, 677. 10485778 10.1097/00000542-199909000-00019

[40] E. H. Stanton , N. D. A. Persson , R. S. Gomolka , T. Lilius , B. Sigurethsson , H. Lee , A. L. R. Xavier , H. Benveniste , M. Nedergaard , Y. Mori , Magn Reson Med. 2021, 85, 3326. 33426699 10.1002/mrm.28645

[41] A. Müller , P. Fries , B. Jelvani , F. Lux , C. E. Rübe , S. Kremp , P. Giovanoli , A. Buecker , M. D. Menger , M. W. Laschke , F. S. Frueh , Investigative Radiology. 2017, 52, 725. 28678084 10.1097/RLI.0000000000000398

